# Role-play Games (RPGs) for Mental Health (Why Not?): Roll for Initiative

**DOI:** 10.1007/s11469-022-00832-y

**Published:** 2022-05-11

**Authors:** Ian S. Baker, Ian J. Turner, Yasuhiro Kotera

**Affiliations:** 1 School of Psychology, University of Derby, Kedleston Road, Derby, DE21 1GB UK; 2 Provost, Learning & Teaching: Student Performance, University of Derby, Kedleston Road, Derby, DE21 1GB UK; 3 University of Nottingham, Nottingham, NG7 2RD UK

**Keywords:** Dungeons & Dragons, D&D, Mental health, Applications, COVID-19

## Abstract

COVID-19 has impacted the mental health of the general public negatively, associated with preventative measures, restricting life activities. These restrictions, such as the stay-at-home strategy, resulted in heightened stress, depression, loneliness, substance abuse, and domestic violence, violating people’s occupational and personal lives. During the pandemic, the demands for role-play games (RPGs) have increased: for example, the sales of “Dungeons & Dragons” tripled, underscoring the potential mental health benefits of such games. However, research into the mental health benefits of such games remains under-developed, needing more scientific attention. Accordingly, this commentary paper reviews the existing literature, and suggests areas for application and research about RPGs and mental health including psychotherapy, career guidance, education, and people with disabilities. Insights offered can help practitioners and researchers in RPGs and mental health conduct empirical research and develop alternative approaches for mental health in stressful times.

## Mental Health Difficulties During COVID-19

The COVID-19 pandemic has brought negative impacts on our mental health due to drastic lifestyle changes (e.g. social distancing, quarantine, lockdown). Derivative impacts such as unstable employment and access to health services as well as the fear of being infected with the virus have raised stress levels (Bhandari et al., 2021). The rates of anxiety and depression are increasing more rapidly than ever before, with a rise of 20% in stress levels since the beginning of the pandemic (Rajkumar, 2020; Vindegaard & Benros, 2020). Levels of domestic abuse and substance use have also increased (Abramson, 2021; Evans et al., 2020). Research has identified many risk factors for mental health difficulties in COVID-19, including poor sleep quality (Liu et al., 2020), being female (Liu et al., 2020), working in healthcare (Kotera et al., 2021), those with a pre-COVID medical diagnosis (Barber et al., 2020), and being a migrant (Bhandari et al., 2021). The diversity of these factors implies that COVID-19 has impacted our mental health on a broad level: many of us have been mentally impacted by COVID-19.

Despite the large and broad impact of mental health difficulties, appraisal for effective treatment remains to be refined. Indeed, reduced rates of mental health difficulties have been found in some countries; however, these changes can be explained by external events. For instance, in the UK, the level of anxiety has dropped from 62% in March 2020 to 42% in February 2021 among the general population, and from 67% in June 2020 to 58% in February 2021 among people with a pre-existing mental health diagnosis (Mental Health Foundation, 2021). These improvements can be understood as being a result of the administration of the vaccine, initiated in December 2020 (British Broadcasting Corporation, 2020) earlier than most countries, but this left the root cause of the COVID-19 mental health difficulties untreated. Unsurprisingly, the rate of people who feel that they are coping with stress associated with the pandemic has reduced from 73% in April 2020 to 64% in February 2021 (Mental Health Foundation, 2021). Significant post-COVID-19 mental health challenges are expected (Mayne, 2020), with the potential for us to experience a worldwide outbreak of mental health problems alongside or following COVID-19 (Pies, 2020). These findings illustrate the need for developing an effective and practical mental health strategy. On an individual level, one emergent coping strategy during the pandemic was the increased use of role-playing games (RPGs).

## Increasing Demand for Role-playing Games (RPGs) During the Pandemic

Role-playing games emerged from wargames which, in turn, originated in ancient strategy games, particularly chess. Role-playing games are unlike wargames in that they are co-operative in nature and the operational level is on the individual rather than groups, units, battalions, etc., and the interactions can be complex, going beyond combat. A typical role-playing game involves a “Dungeon Master” (DM, or “Games Master”) who describes what the other players can see and experience, outlines the nature of the imaginary world, and controls the non-player characters (NPCs) that the players interact with. The DM also outlines the missions, tasks, problems, and challenges the players face, but the decisions they make can be relatively open-ended, resulting in a complex and multi-layered experience. The players each control one (occasionally more) character that has set characteristics and abilities that are determined by a numerical value, and this is supplemented by equipment that may help their abilities. These characteristics and abilities are typically constructed via set formats, and when a player wishes their character to perform an action (e.g. climb a wall, swing a sword, cast a spell, persuade someone), they have to roll a die and add or subtract values based upon how good their character is at that task; typically a higher roll will indicate success, and the DM will describe the consequences of the success or failure of the die roll. This creates a rich, narrative-based interaction between DM and players that is akin to modern storytelling; the players are not playing “against” the DM, but rather all participants are joined together in a shared story experience. This can be face-to-face or online, and there are dedicated online systems (e.g. D&D Beyond, Roll20) for playing such games. Much of the experience relies upon the imaginations of the participants, but this can be augmented with the use of miniatures that represent the players and the NPCs, and two-dimensional maps (with or without three-dimensional terrain) to help make the experience more immersive. In the online environment, this has been extended to players being only able to see the parts of their environment that their characters themselves can see, resulting in surprises for the uncautious (or unlucky) player.

Due to the plethora of games available today, the setting of a particular role-playing game can be varied, from fantasy to science-fiction, from history to horror. The first role-playing game (known as “Generation 1”) available commercially was Dungeons & Dragons (or D&D/DnD), designed by Gary Gygax and Dave Areson and published in 1974.

Although its popularity has ebbed and flowed over the years, it has also experienced its controversies, for example, in the 1980s it formed part of the “Satanic Panic” in the USA which claimed that “this imaginary game could induce players to believe its fantasy was real, they believed that players mistakenly thought the fantasy of the game was imaginary, when it actually indoctrinated them into real-world witchcraft and demonology.” (Peterson, 2021; p. 181) which led to devils and demons being removed from its 2nd Edition (Grundhauser, 2016). There have also been issues with stereotypes around the portal of race with supplements such as Oriental Adventures in 1985 and the Tomb of Annihilation in 2017 being criticised for how Black culture is presented and amalgamated (D’Anastasio, 2017); Garcia (2017) has explored how players’ identities and experiences are influenced by the deceptions of constructs like race and gender in D&D. However, recent research into whether people view orcs as racist has indicated that playing D&D was not associated with higher levels of ethnocentrism or racism, and priming people questions regarding if certain material is racist may prime them to see racism in such material (Ferguson, 2022). Hollander (2021) argues that D&D can yield a personal-political edification providing an informal “moral training” but notes it can also reinforce harmful associations that exist towards others.

Even with controversies, it remains popular. As of 2014, it is now in its fifth edition, and it has seen a tremendous growth in recent years, with Wizards of the Coast (a division of Hasbro which owns the game) estimating over 50 million people had played the game, and their revenue increased 24% in 2020 to over $816 million. Part of this surge in growth has been as a reaction to the COVID-19 pandemic, with Fandom (the owners of D&D Beyond, an online platform where D&D can be played) reporting their number of subscribers doubling between 2019 and 2020, and individuals reporting that playing D&D has supported their mental health during the pandemic (Skipwith, 2021). Although there are a multitude of RPGs available (including highly related ones, such as Pathfinder) and the conclusions drawn here could apply to most of them, due to its historical status in the community and increased popularity during the pandemic, D&D is the main focus of this piece.

## Impact of Role-playing Games on Mental Health

D&D has a long history with mental health. In its early years, it was incorrectly linked by the mainstream media to teenage delinquency and immorality, without any demonstrable causal relationship. Not long after the game’s development, the alleged role of D&D in the disappearance of James Dallas Egbert III and the suicide of Irving Bink Pulling II, alongside religious condemnation of D&D based on its purported links to satanism and other controversies mentioned previously, created a stigma associated with D&D that persists at times today (Kelly, 2019; Sidhu & Carter, 2020; Polkinghorne et al., 2021). This link between D&D and psychopathology, caused by the persistence of these stereotypes, has been investigated by several scholars (Ben-Ezra et al., 2018; Lis et al., 2015). However, their findings suggest that engagement with D&D is not associated with a sign of psychopathology. They furthermore show that familiarity with D&D leads to a lower likelihood of that belief. In fact, regular D&D players demonstrated no difference on the scores in the Beck Depression and Eysenck Personality Inventories compared with a control group, who did not play the game (Carter & Lester, 1998). Additional research examining the impacts, both positive and negative, of D&D is needed.

## Role-play Games in the Clinical Setting

Role-play in clinical practice is reported to be associated with higher levels of reflection, empathy, insights about the client, and peer learning (Rønning & Bjørkly, 2019). By simulating a real situation, participants are more able to appreciate people in the context, leading to better understanding (Caltabiano et al., 2018). RPGs are sometimes used as therapeutic tools in psychodrama and drama therapy; psychodrama therapy involves patients under supervision dramatising a number of scenes such as specific happenings from the past, often with help from a group, enabling them to reflect on and explore alternative ways of dealing with them (Kedem-Tahar & Felix-Kellermann 1996). Drama therapy has more of an emphasis on spontaneity and creativity and employs playful approaches. More specifically, it is a systematic and controlled approach that uses dramatic action to explore emotional issues (Kedem-Tahar & Felix-Kellermann 1996).

It has been suggested that D&D, with its narrative structures about overcoming adversity and exploring alternative identities, mirrors aspects of mental health recovery (Kerr et al., 2020). The use of D&D in a clinical setting is reported as early as 1994 where Blackmon describes the treatment of Fred (19) who suffered from an obsessional, schizoid personality. In the therapy sessions, Blackmon (1994) gradually allows Fred to describe his D&D experiences as he realises it is almost a form of self-therapy and Fred’s character has provided an outlet for working through his emotions in a safe way. Blackmon specifically notes that D and D rule systems shape the fantasy construct of the game, the structure is not there to constrict but to reduce anxiety and enable. Kallam (1984) observed that a group of “mildly handicapped” adolescents who played D&D twice a week, for 9 weeks, gradually developed higher self-efficacy and capacity for creative and complex situations when playing D&D compared to a control group. There are other published studies that touch on the effectiveness of psychodrama and drama therapy adaptations of role-playing, though not in all cases focusing specifically on D&D (Enfield, 2007; Rosselet & Stauffer, 2013; Zayas & Lewis, 1986; Hughes, 1988). Much of this work is based on localised case studies which makes it difficult to extract the essential aspects into clinical recommendations which could be tested on a larger scale. It does indicate that RPGs could provide a structured “safe space” (Dare et al., 2021) for the exploration of psychopathologies.

Henrich and Worthington (2021) conducted a rapid evidence assessment of the therapeutic utility of D&D, and they found a tentative link between D&D and psychological benefits such as creativity (Chung, 2013) and empathy (Rivers et al., 2016). This is supported by the broader literature on RPG, where evidence exists for a wider range of skills positively impacted by RPG. For example, Abbott et al. (2022) have shown that D&D in a small therapeutic setting can help increase confidence, the ability to confront situations, and cope with unexpected events. Furthermore, the author demonstrates that these skills were transferred to the real world.

Polkinghorne et al. (2021) agree RPGs offer great potential in the context of narrative therapy for building resilience and improving the resilience of participants. However, they argue that not enough consideration has been given to the game’s environments and rules themselves, with focus localised upon commercial games like D&D. The collaborative nature of D&D and its scaffolded storytelling experience offers great therapeutic potential but they argue the game *itself* is part of that therapeutic experience. The D&D game world is often used without significant adaptation to the client: there is a lack of exploration of its specific contribution to the therapeutic experience. In a similar theme of tailoring the D&D experience appropriately and the potential benefits of the experience itself, Mendoza (2020) argues that D&D is not for novices and that the games itself contains very little guidance on active role-play (5th Edition) and the games’ rules focus heavily on the “hack-and-slash” mechanisms for gaining in-game experience points to improve your character. They believe the therapeutic benefits come from D&D familiarity, scaffolding, and structured rules that specifically reduce anxiety of choice paralysis. Therefore, appropriate introduction to such games is essential, ideally by experienced and compassionate players.

## Role-play Games in the Non-clinical Setting

The use of role-play games (rather than therapeutic role-play) in a clinical setting could be a valuable tool for clinicians. However, their potential benefits in non-clinical settings show broader promise of assisting people in a COVID-19 world and beyond. Previous studies have been limited in number and focussed on small samples with qualitative approaches, but researchers have studied the benefits of D&D play on mental health and wellbeing in non-clinical settings.

Adams (2013) analysed the Facebook chat log of a nine-person D&D group over a 3-year period using Bormann’s (1972) theme analysis. The four themes that emerged were (1) democratic ideologies, (2) friendship maintenance (3) extraordinary experiences, and (4) good versus evil. The findings provide a description of group members’ real-world needs being met through symbolic in-game interactions evidenced by communicative markers.

In a similar study, Sargent (2014) undertook a qualitative analysis of six D&D players, using uniform narrative analysis from structured interviews. Although specific measures of players’ mental health were not recorded, Sargent did find that playing D&D decreased players’ social anxiety and allowed them to interact with their own emotional content in an experience that “meant something” and often was only (perceived) to be possible in a fictional world. Five of the players reported how trauma during childhood was in part successfully navigated through playing D&D; likewise, the players describe how gaming experiences helped them cope with internal conflicts and stress. The sense of community and belonging was important to the players; they felt that experimentation through their characters led to improved real-world social skills and the development of new relationships. These relationships in turn were reported by the players as helping them cope with stressful situations.

In a qualitative study that focussed more specifically on how D&D relates to mental health, Causo and Quinlan (2021) utilised narrative enquiry to understand the experiences of thirteen players who were recovering from mental health difficulties. Narrative enquiry of the interview transcript was undertaken inductively and deductively, and several themes were identified that linked D&D to their well-being. These were (a) experiencing through characters, (b) skill building, (c) finding space from mental health symptoms, (d) safely engaging with mental health difficulties, and (e) building relationships. The authors report that all five stages of the Psychological Recovery Model (Andresen et al., 2003) were observed and that all players engaged with D&D in a manner reflective of their mental health recovery process.

In a theoretical exploration of how RPGs can benefit individuals, Hall (2015) argues that players benefit from an increased emphasis on role-play as an important aspect of psychosocial growth. The author suggests D&D characters are well-formed, semi-independent personae of their players, who have potential to be equal in influence to an individual’s other expressions of personality. Hall argues that players’ D&D characters, like all aspects of personality, exist at the junction of mythical, psychological, and sociological forces and therefore potentially an important tool for strong mental health. More recently, and particularly in response to the COVID-19 pandemic, there have been several anecdotal accounts in the media of the benefits of playing D&D in supporting the mental health of children (Krieger, 2021) and adults (Hughes, 2021), addressing social anxiety in “post-lockdown angst” (Hazel, 2021) and helping autistic individuals constructively interact with others (Leach, 2021). These qualitative studies and anecdotal reports all provide evidence that playing RPGs such as D&D can aid in friendship and relationship maintenance, mitigation of social anxiety, improved social skills, reducing stress, alleviation from mental health challenges, and providing connection with others. This alone provides strong support for the need for more systemic and wide-scale research into the benefits of playing RPGs in a COVID and post-COVID world.

## Suggestion for Applications and Research

We have several recommendations for the exploration of how RPGs can be researched and used to support mental health:

- Building upon the qualitative research above, further research could be conducted examining the experiences of players during the pandemic and how it had supported them.
- There is a dearth of research looking at larger samples of players, their experiences of and attitudes towards RPGs, and how they have been affected by the pandemic. In addition, how these relate to other personality characteristics such as anxiety, extraversion, relationship formation and maintenance, and others.
- Qualitative approaches could also be used to examine how neurodiverse individuals may be able to experience and interact with others using role-play games as a structured medium of expression.
- Mixed method approaches could examine the types of characters players create and how they use them as a method of exploring aspects of one’s self as a form of positive expression, such as gender identities or the use of archetypes.
- The use of RPGs could be used as an intervention-based approach for the improvement of mental health, such as reducing levels of depression, stress, anxiety, or loneliness.
- In terms of pedagogic research, RPGs could be examined as a game-based learning method for the teaching of probability theory, basic statistics, and exploring ethical dilemmas.
- Due to some of the controversies surrounding the playing of RPGs, when tailoring the experience of playing it (e.g. Polkinghorne et al.’s (2021) suggestion) in the research and applications outlined above, an inclusive environment should be engendered to support the mental health and/or learning of players.

Considering the ever-increasing demands of RPGs associated with the pandemic, their effects on mental health need to be empirically evaluated, as mental health is a cause of concern for many countries today. Insights from this article can help practitioners and researchers in RPGs and mental health conduct empirical research, and develop non-clinical approaches to improve people’s mental health in stressful times. The title of this piece includes the phrase “roll for initiative”, which in D&D is where each player rolls a die to determine the order in which they act in combat, with a high roll going first; it is time for us to roll high and systematically explore how RPGs can help support mental health.
